# Design, synthesis and evaluation in an LPS rodent model of neuroinflammation of a novel ^18^F-labelled PET tracer targeting P2X7

**DOI:** 10.1186/s13550-017-0275-2

**Published:** 2017-04-04

**Authors:** Enrico Raffaele Fantoni, Diego Dal Ben, Simonetta Falzoni, Francesco Di Virgilio, Simon Lovestone, Antony Gee

**Affiliations:** 1 Department of Imaging Sciences and Biomedical Engineering, King’s College London, St Thomas’ Hospital, 4th floor Lambeth Wing, SE1 7EH London, UK; 2 School of Pharmacy, Medicinal Chemistry Unit, University of Camerino, via S. Agostino 1, 62032 Camerino, MC Italy; 3 Prof Francesco Di Virgilio and Dr Simonetta Falzoni, Dipartimento di Morfologia, Chirurgia e Medicina Sperimentale, Sezione di Patologia, Oncologia e Biologia Sperimentale, University of Ferrara, Ferrara, Italy; 4 Department of Psychiatry, Warneford Hospital, University of Oxford, Warneford Lane, Oxford, OX3 7JX UK

**Keywords:** P2X7, F-18, LPS, EFB, Radiosynthesis, Molecular modelling

## Abstract

**Background:**

The P2X7 receptor has been shown to play a fundamental role in the initiation and sustenance of the inflammatory cascade. The development of a novel fluorine-18 PET tracer superior and with a longer half-life to those currently available is a promising step towards harnessing the therapeutic and diagnostic potential offered by this target. Inspired by the known antagonist A-804598, the present study outlines the design via molecular docking, synthesis and biological evaluation of the novel P2X7 tracer [^18^F]EFB. The tracer was radiolabelled via a three-step procedure, in vitro binding assessed in P2X7-transfected HEK293 and in B16 cells by calcium influx assays and an initial preclinical evaluation was performed in a lipopolysaccharide (LPS)-injected rat model of neuroinflammation.

**Results:**

The novel tracer [^18^F]EFB was synthesised in 210 min in 3–5% decay-corrected radiochemical yield (DC RCY), >99% radiochemical purity (RCP) and >300 GBq/μmol and fully characterised. Functional assays showed that the compound binds with nM *K*_i_ to human, rat and mouse P2X7 receptors. In vivo, [^18^F]EFB displayed a desirable distribution profile, and while it showed low blood–brain barrier penetration, brain uptake was quantifiable and displayed significantly higher mean longitudinal uptake in inflamed versus control rat CNS regions.

**Conclusions:**

[^18^F]EFB demonstrates strong in vitro affinity to human and rodent P2X7 and limited yet quantifiable BBB penetration. Considering the initial promising in vivo data in an LPS rat model with elevated P2X7 expression, this work constitutes an important step in the development of a radiotracer useful for the diagnosis and monitoring of clinical disorders with associated neuroinflammatory processes.

**Electronic supplementary material:**

The online version of this article (doi:10.1186/s13550-017-0275-2) contains supplementary material, which is available to authorized users.

## Background

The P2X7 receptor has been identified as a key player in the neuroinflammatory cascade leading to the onset and progression of a wide range of CNS conditions [1]. For example, in Alzheimer’s disease, it is crucial in mediating the secretion of interleukin-1β following beta-amyloid stimulation [2]. Likewise, neuropathic pain is directly related to P2X7’s action on IL-1β maturation [3] and cathepsin release [4]. Therefore, the development of a non-invasive selective PET ligand targeting P2X7 would enable further understanding of the role of this important receptor, as well as providing a measurable outcome for clinical trials and providing a new avenue for the diagnosis of neuroinflammation.

**Fig. 1:**
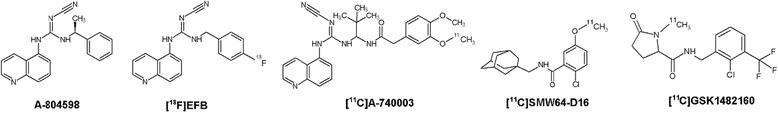
Chemical structures of A-804598 and of the P2X7 PET tracers described to date in the literature, including our novel P2X7 ligand [^18^F]EFB

Over the last decade, a number of PET tracers have been developed and evaluated (Fig. 1). Neither [^11^C]A-740003 nor [^11^C]SMW64-D16 showed appreciable brain uptake; however, both succeeded in recognising the site of inflammation in two different rodent models when examined by in vitro autoradiography [5, 6]. [^11^C]GSK1482160, however, shows greater promise due to its efficient radiosynthesis and strong P2X7 selectivity and blood–brain barrier (BBB) permeability, but its preclinical evaluation is still in progress [7].

This study presents the novel fluorine-18-labelled PET tracer [^18^F]EFB 2-cyano-1-(4-[^18^F]fluorobenzyl)-3-(quinolin-5-yl)guanidine developed from the known blood–brain barrier permeable P2X7 antagonist A-804598 [8, 9]. Cyanoguanidines have been investigated at length due to their strong pharmacological activity and affinity for P2X7 across a number of analogues. This includes the discovery that an *N*-quinoline substituent results in higher binding affinity compounds compared with analogues with smaller or less hydrophilic aromatic rings [10, 11], and that a 1-carbon guanidine-phenyl linker is more desirable than longer and more functionalised analogues [12–14]. Instead, the cyanoguanidine core is thought to provide rigidity to the molecular structure by hydrogen bonding with the receptor-binding pocket [15]. Hence, compound EFB is ideally placed to display an optimal fit into the binding pocket while featuring a fluorine-18 label. The work presented here includes a molecular docking analysis of the structure–affinity relationship (SAR) between the receptor ATP-binding pocket and both the benzyl-desmethylated derivative of A-804598 and compound EFB. The synthesis and radiolabelling of all compounds required for the synthesis of [^18^F]EFB are also described. Finally, the biological evaluation of [^18^F]EFB in P2X7-transfected cells and in an intraperitoneal lipopolysaccharide (LPS) preclinical model of inflammation is presented.

## Methods

### Molecular modelling

All molecular modelling studies were performed on a Core i7 CPU (PIV 2.20 GHZ) PC workstation. Homology modelling studies of the human P2X7 (hP2X7) were carried out using the closed state zebrafish P2X4 (zP2X4) receptor as a template, as described in Dal Ben [15]. Molecular Operating Environment (MOE by C.C.G., Montreal, CA, version 2012.10) suite was employed for this task. The ligand structures were docked into the binding site of the P2X7 receptor using the AutoDock tool (PyrX interface) by the Scripps Institute [16, 17]. All docking conformations were then imported in MOE, and the partial charges were assigned by semi-empirical RHF/AM1 calculations using the MOPAC package [18] implemented in MOE. Further details are provided in Additional file 1.

### Chemistry

**Fig. 2:**
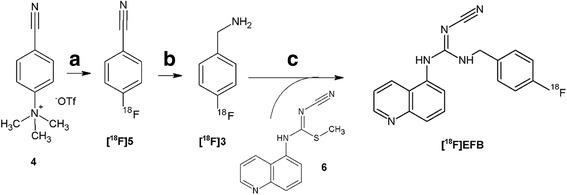
Radiosynthetic route to [^18^F]EFB. *a* [^18^F]KF.K222, 15 min, 110 °C in DMSO followed by tC18 Sep-Pak SPE. *b* 2:1 *w*/*w* NaBH_4_/Co(OAc)_2_ in 2 mL:1 mL THF/H_2_O, 5 min followed by Strata-X-CW purification in acidic media. *c* Excess triethylamine, THF, 140 °C, HPLC purification and concentration on a light C18 Sep-Pak SPE cartridge

Hereafter, the synthesis of precursor 6 and of the reference compound EFB is briefly described (Fig. 2). Full compound characterisation is described in Additional file 1, together with the synthesis of compounds 4 and 2.

For the synthesis of methyl *N*′-cyano-*N*-5-quinolinylcarbamimidothioate (6), a mixture of 5-isothiocyanatoquinoline (2, 6.38 g, 34.3 mmol) and sodium hydrogencyanamide (2.19 g, 34.3 mmol) in dimethylformamide (30 mL) was stirred at room temperature for 1 h. Methyl iodide (2.13 mL, 34.3 mmol) was added at 0 °C, and the reaction was stirred at room temperature for 2 h. The reaction was poured into water and stirred further for 20 min. The orange precipitate was filtered and washed with water. Further purification was performed by flash chromatography on SiO_2_ (EtOAc:CH_2_Cl_2_, 1:1) to obtain methyl *N*′-cyano-*N*-(quinolin-5-yl)carbamimidothioate (compound 6) as an orange solid in 70.0% yield (5.77 g).

In line with previously published procedures [8], for the synthesis of 2-cyano-1-(4-fluorobenzyl)-3-(quinolin-5-yl)guanidine (EFB), a mixture of 0.62 mL 4-fluorobenzyl amine 3 (5.36 mmol, Alfa Aesar) and 1.12 mL triethylamine (8.04 mmol) was added to compound 2 (1 g, 5.36 mmol) in 25 mL anhydrous tetrahydrofuran (THF) and stirred for 1 h at room temperature. Following the addition of mercury acetate (5.36 mmol) and sodium hydrogen cyanamide (16.1 mmol), the mixture was stirred for 24 h until dark black. The mixture was filtered under reduced pressure through a short pad of celite and washed with acetone (30 mL). The filtrate was concentrated by rotary evaporation and purified by flash chromatography (SiO_2_, 8–10% MeOH in Et_2_O, then 0–25% EtOAc in 10% MeOH in Et_2_O). The product was then dried by rotary evaporation and recrystallized from acetone as a white powder in 8% yield (140 mg).

### Radiochemistry

#### Chromatography

Chemical and radiochemical purities were assessed by analytical radio- and UV-HPLC as outlined in Table 1. The chemical identity of [^18^F]EFB was confirmed by comparison with its respective standard reference compound, while intermediate radioactive species were confirmed by thin-layer chromatography (TLC). The molar radioactivity was determined via a standard UV curve with a sample of the product in its final formulation. All radiochemical yields (RCY) are reported as decay-corrected values.

**Table 1:** HPLC methods

Method	EFB Rt (min)	Flow rate (mL/min)	Column	Mobile phase gradient	Mobile phase	UV (nm)
A	51’10”	3	Agilent eclipse XDB C18, 9.4 × 250 mm, 5 μm, 100 Å	15% B for 5 min; 15–35% B for 1 h; 35% B for 2 min; 35–20% B for 2 min; 20% B for 1 min	A: H2O	254
					B: MeCN	
B	19’10”	1	Kinetex Phenomenex XB-C18 with guard, 4.6 × 150 mm, 5 μm, 100 Å	30 s at 20% B; 23.5 min 20–33.5% B; 3 min 33.5–70% B; 2 min 70% B; 1 min 70–20% B	A: H2O	300
					B: MeCN	

#### Radiosynthesis

[^18^F]Fluoride was produced using an RDS112 cyclotron at King’s College London PET Centre by the ^18^O(*p*,*n*)^18^F reaction via proton irradiation of enriched (95%) ^18^O water. 1–1.5 GBq of aqueous [^18^F]fluoride were trapped in a Sep-Pak Accell Plus QMA cartridge (WAT023525) pre-activated with 5 mL 1 N NaOH, 10 mL water and 5 mL 1 N K_2_CO_3_ followed by 10 mL water. The fluoride was released with 0.65 mL 30 mM:15 mM Kryptofix 222/potassium carbonate dissolved in 85:15 acetonitrile/water. The solution was dried three times at 90 °C for 5 min in a Wheaton V vial and under a flow of nitrogen.

For the synthesis of 4-fluorobenzonitrile ([^18^F]5), the method reported by Koslowsky [19] was followed with some modifications. To dried [^18^F]fluoride (1–1.5 GBq), 4-cyano-*N*,*N*,*N*-trimethylanilinium trifluoromethansulfonate 1 (2 mg) in DMSO (0.3 mL) was added and reacted for 15 min at 110 °C. Once the reaction was completed, the mixture was diluted with water (30 mL) and passed through a Waters Sep-Pak tC18 Plus Long SPE cartridge (WAT036800). The cartridge was washed with additional water (5 mL) before elution of compound [^18^F]5 in 2 mL THF in 40–60% RCY and >95% radiochemical purity (RCP). Radio-TLC (SiO_2_, petroleum ether/ethyl acetate (1:1), R_f_ 0.7; lit. [19] 0.7).

For the synthesis of 4-fluorobenzyl amine ([^18^F]3), 2 mL [^18^F]5 in THF was transferred without further purification into a reaction vial containing 10 mg NaBH_4_ and 5 mg Co(OAc)_2_. To this, 1 mL water was added and the mixture was mixed and allowed to react at RT for 3 min. Next, the mixture was diluted in 40 mL pH 6.2 phosphate buffer (6.7:1 1 M NaH_2_PO_4_:1 M Na_2_HPO_4_, *I* = 9 mM) so as to limit the organic solvent to 5%. The mixture was filtered (0.2-μm pore Whatman Spartan filters) and loaded on a Phenomenex Strata-X-CW SPE cartridge (8B-S035-FBJ). Following a 5 mL acetonitrile wash, a purple band characteristic of compound [^18^F]3 formed in the cartridge and could be entirely eluted in the second of three aliquots of 0.6–0.7 mL 5% TFA acetonitrile in 45–55% RCY (22–34% overall RCY) and >93% RCP. Radio-TLC (SiO_2_, nBuOH/HOAc–H_2_O (4:1:1), *R*_f_ = 0.4; lit.[19] 0.4).

Finally, for the synthesis of [^18^F]EFB, 0.4–0.7 mL of [^18^F]3 in 5% TFA acetonitrile were neutralised with 2 eq. triethylamine (110–112 μL) and added to 11 μmol of compound 6 (3.6 mg) in a sealed vessel. The reaction vessel was heated at 160 °C for 15 min, cooled to 50 °C and the solvent was concentrated at 50 °C under a stream of nitrogen for 7–10 min. Next, the crude mixture was diluted in 2 mL water ensuring <15% acetonitrile was present and injected in an HPLC semi-prep C18 column to provide isolation of the pure product in >99% RCP and >300 GBq/μmol molar radioactivity. The product was diluted to 30–50 mL in water and passed through a light C18 Short Sep-Pak SPE cartridge (WAT023501) for concentration in 200–500 μL ethanol. The resulting product was obtained in high purity and 4.0% overall RCY in 3 h and 30 min from start of synthesis. Purity tests were performed with HPLC method B (Table 1). A stability test performed in 0.05% DMSO at 37 °C for 2 h indicated no radioactive by-product formation and high retention of its UV-active fingerprint (254 nm, Additional file 1: Figure S1).

#### Optimisation of the reaction conditions

Iterative optimisation steps were performed with the use of 100–150 MBq starting [^18^F]fluoride and were aimed at improving the radiochemical yield of each step of the radiosynthesis. The influence of the following parameters was explored by TLC (compounds [^18^F]5 and [^18^F]3) and HPLC analysis ([^18^F]EFB) of 50 μL reaction mixture samples filtered and diluted to 200 μL in water:

- Temperature
- Precursor concentration
- Time
- Purification technique
- Catalytic base concentration

### In vitro affinity assays

The materials and methods employed in this section are described in full in Cabrini 2005 [20] and in Additional file 1. Briefly, HEK293 were transfected with the calcium phosphate method described in Rizzuto [21] and Morelli [22]. Stably P2X7-transfected, single-cell-derived clones were obtained by limiting dilutions. Transfected cells were then kept under selection in the presence of 0.2 mg/ml G418 sulfate. Instead, B16 cells natively expressed the mouse P2X7 receptor. Experiments were performed in a Ca^2+^-containing (1 mM) saline solution (150 mM NaCl, 5 mM KCl, 1 mM MgCl, 5.5 mM glucose, 20 mM Hepes, pH 7.4) at 37 °C with a PerkinElmer fluorimeter. Cells were loaded with 1 μM of the fluorescent indicator Fura-2/AM for 30 min in the presence of 250 μM sulfinpyrazone to reduce excretion of intracellular Fura-2. Cells were then rinsed and resuspended in saline solution at a final concentration of 500,000/ml [23]. Excitation ratio and emission wavelengths were 340/380 and 505 nm, respectively. The calcium ionophore ionomycin (1 μM) was added at the end of each time course as an internal assay control. Inhibitory activity was measured as intracellular Ca^2+^ ([Ca^2+^]_i_) flux variation from baseline while in the presence of 100% activating BzATP (100 μM for hP2X7 and rP2X7, 200 μM for mP2X7). Inhibition was measured on both peak and plateau phases. The final value was chosen as the most accurate of the two measurements, or alternatively from the plateau. A-740003 was purchased from TOCRIS Bioscience.

### Preclinical

#### Animals

Male Sprague–Dawley rats of 240–270 g were bought from Charles River Laboratories International, Inc., and used within 90 days. The animals were housed with a week of acclimatisation before procedures were done under pathogen-free conditions in a temperature- and humidity-controlled environment and given access to food and water ad libitum. All experiments were carried out in the Rayne Institute, St. Thomas’ Hospital, King’s College London, between 2015 and 2016 and according to the Animals Act 1986 and with the approval of the King’s College research ethics committee.

#### Injections

Twenty-four hours prior to scanning, all rats were pre-treated by an intraperitoneal injection of 0.5 mg/kg LPS from *Escherichia coli* (0111:B4 L2630, Sigma Aldrich) at 0.5 mg/mL or with 1 mL/kg sterile saline solution and weighed before the start of the PET imaging procedure. Then, the saline-dissolved reformulated radiotracer was injected under isoflurane anaesthesia in 0.2–5 MBq, corresponding to an administered dose of 0.5–16 ng/kg, and >300 GBq/μmol molar radioactivity via tail vein cannulation, followed by a 0.15 mL saline flush. Details on anaesthesia and PET/CT acquisition and analysis are reported in Additional file 1. Immediately after imaging, the animals were culled by terminal anaesthesia and cervical dislocation and dissected. Organs and body fluids were collected for gamma counting (LKB Wallac 1282) and biodistribution analysis.

#### Statistical analysis

Data are expressed as mean ± standard error mean (SEM) from three independent replicates, unless otherwise indicated. Statistical analysis of the %ID/g variation in the PET scans and biodistributions were performed with GraphPad Prism 5.0 (San Diego, USA) as a parametric two-way ANOVA test with Bonferroni post-test, while the mean longitudinal percentage injected dose per gram (%ID/g) and the post-LPS treatment weight variation were analysed with a Student’s *t* test. The asterisk indicates *P* < 0.01; **P < 0.005; ***P < 0.001. Where unspecified, no significance was found.

## Results

### Molecular docking

**Fig. 3:**
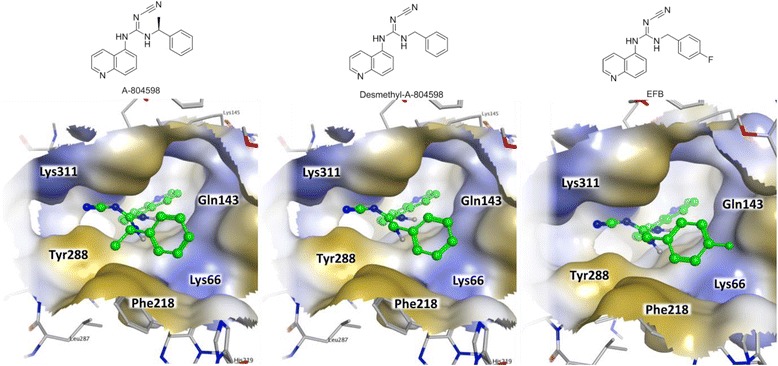
Molecular docking of compounds A-804598 (*left*), its benzyl-desmethylated derivative (*middle*) and EFB (*right*) into the hP2X7 receptor ATP-binding pocket

Compound EFB as well as A-804598 and its benzyl-desmethylated derivative were docked at the hP2X7 binding site, and the docking conformations of the three compounds are shown in Fig. 3.

The removal of the benzyl methyl group of A-804598 only marginally affected the strength of ligand–receptor docking scores due to the decreased interaction with the hydrophobic pocket between Y288 and F218 (Table 2). Nevertheless, it did not alter the three-dimensional conformation of the substrate if not by reducing the structural constraint on the external phenyl group to sit between F218 and K66, leading to minor losses in docking scores. These were not substantially modified either by the insertion of a *para*-fluorine atom useful as a radiolabel, which appeared to comfortably sit at the outer opening of the pocket.

**Table 2:** Post-docking affinity analysis

Compound	Binding_energy (kcal/mol)	Dock_p*K*_i_
A-804598	−6.31	5.14
Desmethyl-A-804598	−5.92	4.87
EFB	−5.72	4.90

### Chemistry

**Fig. 4:**
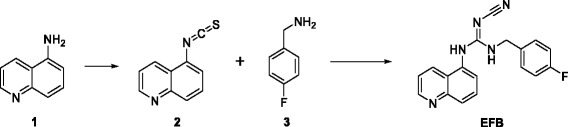
Synthetic route to EFB

The synthesis and characterisation of precursors 4 and 6 as well as of the reference compound EFB (Fig. 4) were successfully carried out. The synthesis of 6 and EFB involved two steps starting from 5-aminoquinoline and was achieved in 43.4 and 5.6% overall yields, respectively.

### Radiochemistry

The radiofluorination procedure loosely followed methods previously reported by Koslowsky [19] and Turkman [24]. The protocol, described in the materials and methods, was modified to increase the radiosynthetic reliability by iterative reaction parameter modifications and alterations to the purification strategies.

#### Optimisation of the reaction conditions

Firstly, in order to minimise the cold carrier reactants in the reaction pot as a means to achieve high molar radioactivities, the procedure for the synthesis of [^18^F]5 was modified to suit 1–2 mg of precursor (3.2–6.4 μmol). Sequential modification of the time and temperature of reaction enabled to conclude that 6.4 μmol precursor reacted with [^18^F]fluoride at 110 °C for 15 min afford a maximal RCY of 75.9 ± 4.7%, superior to that described elsewhere [19] (Table 3).

**Table 3:** Iterative n.i. RCY optimisation of compound [^18^F]5. Values derived from TLC chromatogram analysis

Entry	Scale (μmol precursor)	Time (min)	Temperature (°C)	RCY (% ± SEM)
1	3.2	15	95	63.0 ± 4.1 (*n* = 8)
2	3.2	20–25	95	65.2 ± 7.4 (*n* = 3)
3	3.2	15	110	42.9 ± 3.7 (*n* = 2)
4	6.4	15	95	68.0 ± 17 (*n* = 2)
5	6.4	15	110	75.9 ± 4.7 (*n* = 4)
6	6.4	20	110	66.9 ± 3.2 (*n* = 2)

The nitrile reduction of [^18^F]5 to [^18^F]3 was monitored by TLC ([^18^F]3 *R*_f_ 0.7) at three time points while maintaining the reagent proportions indicated in Koslowsky [19]. As indicated in Table 4, maximal RCYs were obtained with 3 min of exposure of the reagents to a reductive environment.

**Table 4:** N.i. RCY TLC monitoring at different time points for the synthesis of [^18^F]3

Entry	Time (min)	Temperature (°C)	RCY (% ± SEM)
1	1	25	67.1 ± 3.0 (*n* = 3)
2	3	25	90.1 ± 4.1 (*n* = 7)
3	5	25	82.5 ± 5.5 (*n* = 14)

In our hands, the isolation of [^18^F]3 by Waters C18 plus Sep-Pak solid phase extraction in basic media [19] gave poorly consistent product recoveries of <50%. Instead, much higher reliability and up to 60% recoveries were achieved with a Phenomenex Strata-X-CW weak cation exchange cartridge as described in Additional file 1.

**Fig. 5:**
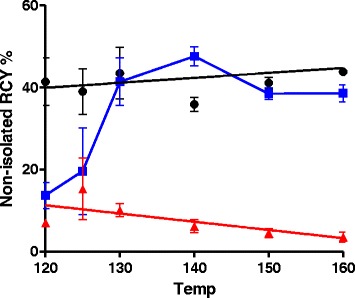
[^18^F]EFB n.i. RCY changes with temperature. *Black*, [^18^F]EFB; *blue*, unknown by-product; *red*, unreacted [^18^F]3

For the synthesis of [^18^F]EFB, iterative variation of the reaction conditions was performed while maintaining 11 μmol of reagent 6 in a 1 mL solution of compound [^18^F]3 in MeCN + 5% TFA in the presence of 2 eq. of triethylamine with respect to TFA. Despite the lack of a statistically significant variation in yield, maximal product formation and near-total reactant consumption was achieved at 160 °C in 15 min, as outlined in Table 5 and Fig. 5.

**Table 5:** HPLC monitoring of the n.i RCY of [^18^F]EFB

Entry	Temp (°C)	[^18^F]EFB RCY (% ± SEM)	Unreacted compound 3 RCY (% ± SEM)	Major by-product RCY (% ± SEM)
1	120	41.4 ± 8.2 (*n* = 2)	7.0 ± 0.8 (*n* = 2)	13.7 ± 4.5 (*n* = 2)
2	125	39.0 ± 7.9 (*n* = 2)	15.3 ± 10.6 (*n* = 2)	19.6 ± 14.9 (*n* = 2)
3	130	43.5 ± 8.9 (*n* = 2)	10.1 ± 2.2 (*n* = 2)	41.4 ± 8.2 (*n* = 2)
4	140	35.9 ± 2.4 (*n* = 2)	6.2 ± 2.2 (*n* = 2)	47.6 ± 3.3 (*n* = 2)
5	150	41.1 ± 2.3 (*n* = 3)	4.5 ± 1.9 (*n* = 3)	38.5 ± 2.5 (*n* = 3)
6	160	43.8 ± 0.8 (*n* = 2)	3.5 ± 1.8 (*n* = 2)	38.6 ± 2.9 (*n* = 2)

#### Purification and quality control of [^18^F]EFB

**Fig. 6:**
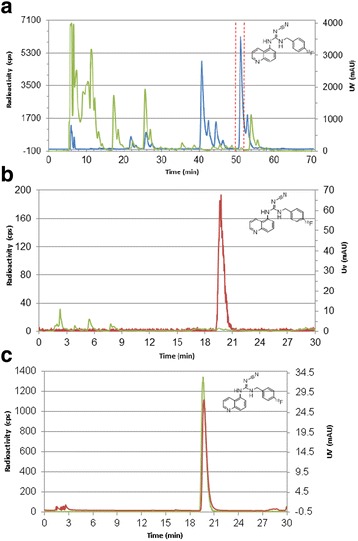
**a** Semi-preparative purification of the product [^18^F]EFB with HPLC method A (Table 1). **b** Final [^18^F]EFB analytical chromatogram (RCP >99%, high CP) with HPLC method B. **c** Identification of [^18^F]EFB by co-elution with the reference standard with HPLC method B. *Green chromatograms*, UV; *blue/red chromatograms*, radioactivity. Only the region marked by the *dashed red lines* around 51 min was subject to collection

The product was purified by semi-preparative chromatography using method A (Table 1, Fig. 6a) and identified by co-elution with an authentic reference compound with method B (Table 1, Fig. 6c). Quality control was performed after purification and reformulation, typically yielding an HPLC chromatogram similar to that shown in Fig. 6b and pH within a physiological range. The desired product [^18^F]EFB had a retention time of 19.35 min.

### In vitro affinity

The affinity of EFB for P2X7 was determined by calcium influx assays in transfected HEK293 and B16 cells and compared with A-740003 affinities (Table 6). For EFB, the assays showed 5.74 pIC_50_ for the human receptor isoform, 5.14 pIC_50_ for rat and 5.22 IC_50_ for mouse P2X7. Assuming competitive inhibition as per A-804598 [8], the values were transformed to their respective inhibition constants (*K*_i_) by means of the Cheng–Prusoff equation [25]. Here, pEC_50_ values for 100% P2X7-activating BzATP (6.8 ± 0.14 in human, 6.3 ± 0.13 in rat and 4.7 ± 0.07 in mouse) were calculated with respect to the YoPro dye uptake response in P2X7-HEK293 cells as reported in Hibell [26]. This resulted in 2.88 nM *K*_i_ in human P2X7, 36.1 nM in rat and 547 nM in mouse. Similar calculations were performed for the known antagonist A-740003, for which literature values useful as internal reference are provided.

**Table 6:** EFB and A-740003 calcium influx inhibition of P2X7 receptor subtypes

P2X7 isoform	Cell line	[BzATP] (μM)	pIC_50_	Lit. pIC_50_	*K*_i_ (nM)	pK_i_
Human	P2X7-HEK293	100	5.74	n/a	2.88	8.54
Rat	P2X7-HEK293	100	5.14	n/a	36.1	7.44
Mouse	P2X7-B16	200	5.22	n/a	547	6.26
Human	P2X7-HEK293	100	7.18	7.36 [32]	0.10	9.98
Rat	P2X7-HEK293	100	5.76	7.75 [32]	8.67	8.06
Mouse	P2X7-B16	200	5.97	6.57 [34]	97.20	7.01

### Preliminary in vivo evaluation

#### LPS model validation

LPS injections are known to induce a 5–10% weight loss within 24 h, which is known to indicate activation of the inflammatory process [27, 28]. In this work, the validation of the LPS rat model was restricted to recording a significant weight loss for all treated rats after 24 h (Additional file 1: Figure S3).

#### [^18^F]EFB evaluation

**Fig. 7:**
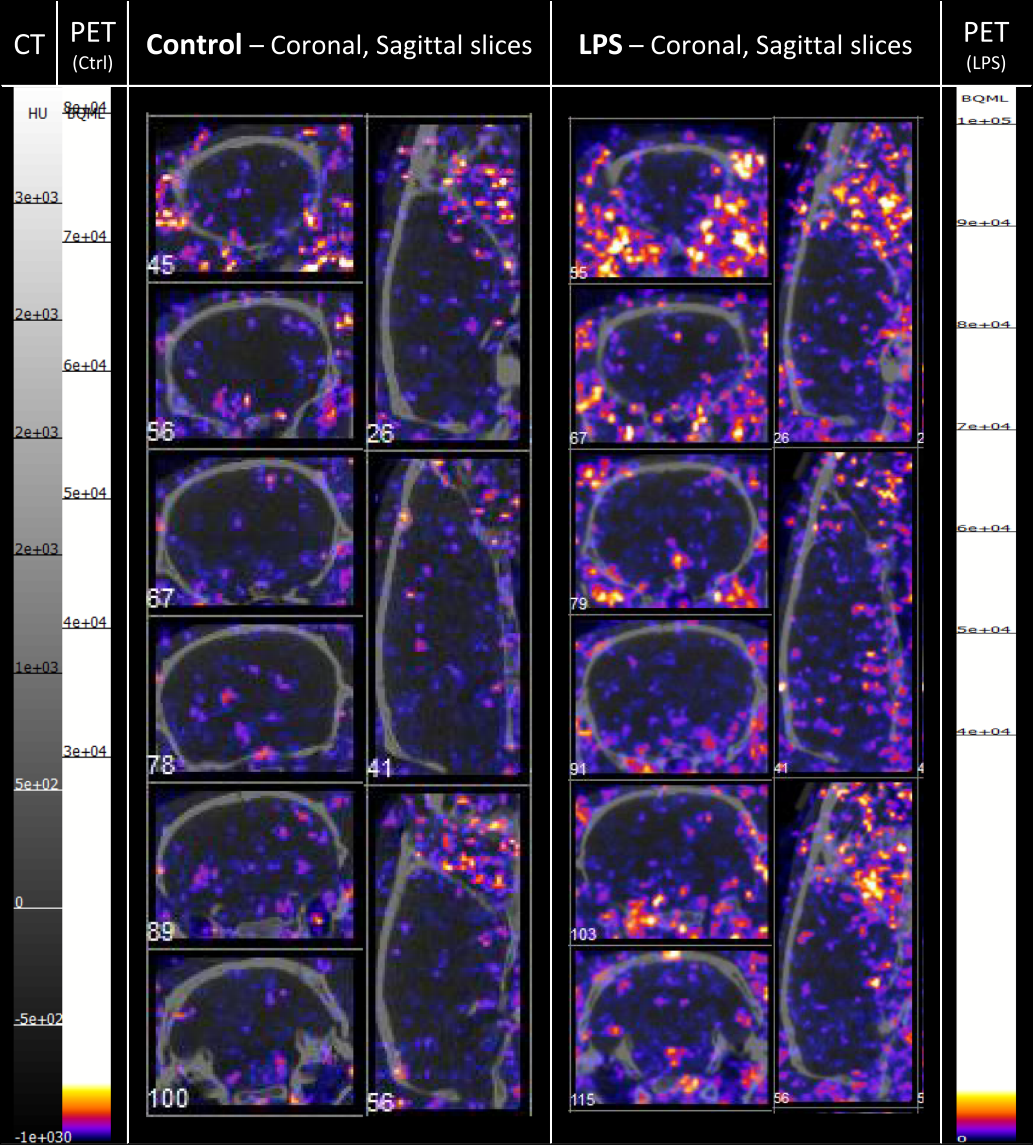
Selected coronal and sagittal tile views of PET/CT scans at 22.5 min in control (ctrl) and LPS male Sprague–Dawley rats injected with comparable doses of [^18^F]EFB. Tile numbers nominally indicate the distance of the slice from the *front* (coronal) and *left* side (sagittal) of the brain, but do not necessarily correspond between different animals. The grey scale in Hounsfield units (HU) refers to the CT data, while the colour scales in Bq/mL refer to the control rat (*left*) and LPS rat (*right*) PET data

**Fig. 8:**
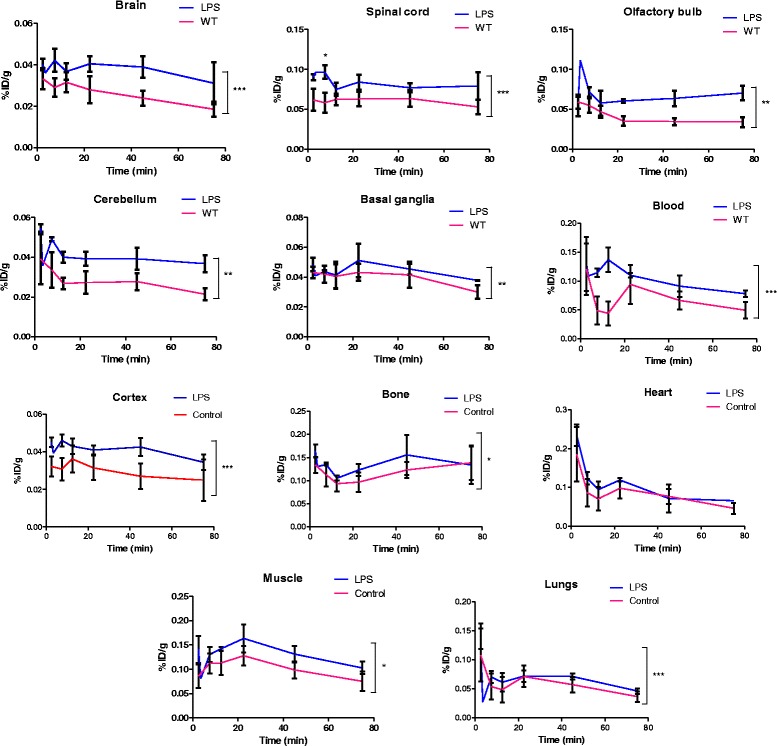
In vivo %ID/g of [^18^F]EFB in 24 h post-LPS injection and control rats (*n* = 3). Matched time and treatment variation significances representative of overall variations in the uptake are reported for each organ. Statistical analysis was carried out by a parametric two-way repeated measures ANOVA with column matching and with Bonferroni post-test. The asterisk indicates *P* < 0.01; **P < 0.005; ***P < 0.001

**Fig. 9:**
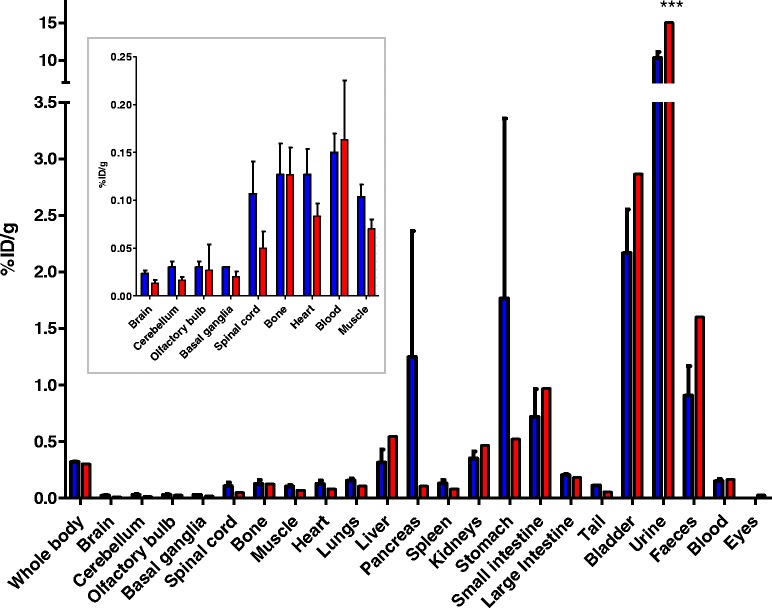
Biodistribution of [^18^F]EFB at 90 min post-injection in LPS (*blue bars*) vs. control (*red bars*) rats. The insert is a blow-up of some regions of interest with low tracer accumulation

Evaluation of [^18^F]EFB as an inflammation-dependent tracer was performed by comparison of control and 0.5 mg/kg LPS-pre-treated Sprague–Dawley rats. The visual PET image assessment indicates low brain uptake with slightly increased retention in the LPS rat’s temporal lobe (Fig. 7, also displaying elevated retinal uptake). Rat longitudinal uptake profiles (Fig. 8) revealed a relatively stable blood concentration around 0.05–0.1%ID/g. The CNS uptake stayed between 0.05–0.1%ID/g for the spinal cord, while it reached 0.02–0.07%ID/g in the whole brain, the cortex, the olfactory bulb, the cerebellum and the basal ganglia. These percentages closely correlate with the terminal biodistribution (Fig. 9). Additionally, LPS-treated rats demonstrated a statistically significant increase in mean tracer uptake compared to untreated rats, possibly reflective of increased P2X7 expression.

## Discussion

The development of the novel radioligand [^18^F]EFB represents a valuable advancement to the field of P2X7 PET imaging. Molecular docking analysis of the benzyl-desmethylated version of A-804598 and of EFB indicated that subtle structural modifications in the periphery of the A-804598 scaffold are not predicted to impact the affinity to the target’s ATP-binding pocket [8], thus offering the possibility of attaching a fluorine-18 label to the externally oriented phenolic ring of A-804598.

The synthesis of EFB was achieved via mercury sulfide precipitation-driven stepwise amine addition in moderate yields, possibly due to interaction of the transition metal with the guanidine moiety. Radiolabelling was achieved in three steps starting from compound 4, achieving an overall DC RCY of 3–5%, >99% RCP and >300 GBq/μmol molar activity. The RCY was mostly limited by the inherently high unknown by-product formation in the final step of the process. This could consist in a cyclised or truncated form of [^18^F]EFB, which could be limited by the replacement of precursor 6 with an analogue with a more labile leaving group, such as sulfonyl methane [29] or sulfonyl chloride [30]. However, some preliminary attempts at oxidising the thiomethyl group with 2 eq. oxone at room temperature or *m*-CPBA at −20 °C resulted in over-oxidation of compound 6. The RCY was found to be sensitive to variations due to limited SPE recoveries unless large crude product dilutions were carried out.

In vitro affinities were derived from calcium influx binding assays in the presence of the calcium-sensitive dye Fura-2/AM in stably P2X7-transfected cells. The typical ATP-mediated Ca^2+^ flow response is a sharp peak followed by a sustained plateau phase [31]. Given the high P2X7 expression, despite the values reported represent the former, the inhibition constants were in a similar range for both the peak and plateau phases. The variation in affinity between the receptor isoforms reflects the trends observed for analogous ligands [32, 33] and indicated a stronger interaction with the human form. This could lead to the underestimation of the sensitivity of this ligand upon clinical translation. The EFB affinity values arising from this assay were found in parallel to those of the known antagonist A-740003, which were compared to literature values arising from similar assay formats [32, 34]. In our in vitro assays, A-740003 appeared less tightly P2X7 binding than apparent from the literature, suggesting that also EFB IC_50_s may result somewhat stronger in different experimental conditions [26]. Moreover, it remains important to clarify if its selectivity to P2X7 differs from its cyanoguanidine analogue A-804598 [8] and to further elucidate whether EFB is truly a competitive inhibitor, as suggested by Donnelly-Roberts [8] and Honore [14] for analogous structures, but recently questioned by Karasawa [35]. A binding modality other than competitive would preclude the use of the Cheng–Prusoff equation in its basic form [36].

A preliminary preclinical evaluation of [^18^F]EFB was carried out with the intention of clarifying its in vivo biodistribution and P2X7 binding and to explore its ability to penetrate the BBB. The use of the bacterial endotoxin lipopolysaccharide (LPS)-inflamed rat model was decided to enable comparison between the tracer uptake under low and high P2X7 expression levels. This is known to induce increased P2X7 expression as demonstrated by immunohistochemical staining of rat brains 24 h after an intraperitoneal 0.5 mg/kg LPS injection [28]. Additionally, LPS-injected rats display protracted microglial activation [37, 38] and raised levels of a range of blood inflammatory markers, including TNF-α, NF-κβ and IL-1β [39]. Moreover, brain cortex expression of IL-1β, IL-6, toll-like receptors (TLRs) and glial fibrillary acidic protein (GFAP) are upregulated within 4 h, suggesting that glial activation and neuronal inflammation quickly follow systemic inflammatory episodes [40, 41], often outlasting them [42]. These biochemical changes are concomitant with an exaggerated weight loss [43, 44], and this measure was employed in this work to demonstrate effectiveness of the LPS treatment. A full validation of the process linking LPS to P2X7 upregulation is ongoing. The 0.5 mg/kg LPS dose was preferred to higher doses based on reports that below 3 mg/kg, the endotoxin does not cause BBB disruption [45].

[^18^F]EFB displays a desirable in vivo distribution and excretion profile in that target tissue retention is quantifiable and that fast removal of the radioactivity predominantly via the urinary route can be observed. The brain uptake of the tracer is quantifiable but low, suggesting that [^18^F]EFB could be a substrate of BBB efflux pumps or that its lipophilicity may benefit from a reduction by scaffold modifications (EFB: LogP 2.72 by miLogP [46]). However, the higher affinity of EFB for the human P2X7 isoform may result in enhanced CNS uptake and retention in a clinical setting.

The mean longitudinal uptake of [^18^F]EFB appears significantly higher in LPS compared to that in untreated rats, although individual time points did not reach significance (Fig. 8). This suggests that the tracer has potential to discern between high and low P2X7 expression in vivo.

The difference in blood tracer quantification values noticeable in Fig. 8 can be a result of partial volume effects arising from the inherently limited dimensions of the blood ROI (the coronary artery lumen), whereas the blood concentration of the tracer resulting from the 90 min biodistribution appears substantially unvaried in the two groups. Furthermore, the 90 min biodistribution data shows an increased, albeit non-significant, tracer uptake in the LPS rats’ upper digestive organs (stomach, pancreas), possibly due to the relative abundance of P2X7 receptors on the epithelial lining of the digestive organs.

The preclinical evaluation was intended as a preliminary assessment of the BBB permeability and overall distribution of the tracer in LPS and control rats. While it was not possible to perform metabolite and kinetic analysis at this stage, further work aiming to explore EFB scaffold variants could benefit from such data. Moreover, the injected activity was modest for some rats, and [^18^F]EFB required extensive synthesis time. This process may benefit from automation or a revised synthetic protocol, such as one including a better precursor leaving group for the final step of the radiosynthetic procedure (step c, Fig. 2).

## Conclusions

The work presented here constitutes an important step towards the development of a fluorine-18-labelled PET tracer useful for imaging P2X7 in vivo. The synthesis and radiosynthesis of [^18^F]EFB have been successfully carried out, affording the tracer in >99% RCP and >300 GBq/μmol molar activity. In vitro calcium influx binding assays revealed a *K*_i_ of 2.88 nM in human P2X7, 36.1 nM in rat and 547 nM in mouse.

A preliminary in vivo evaluation indicated a low BBB uptake indicative of limited suitability of the molecule for P2X7 brain PET imaging. Taken together with previous work by Janssen [6] on a different radiotracer targeting P2X7, this data is suggestive of limited compatibility of the cyanoguanidine moiety with BBB entry in rats. Nevertheless, the altered mean longitudinal uptake in LPS versus control rats indicates that the tracer may have potential as a systemic P2X7 PET imaging agent.

## Additional file

Additional file 1: Supplementary materials. (DOCX 284 kb)
