# Supplementary material for: Design, synthesis and evaluation in an LPS rodent model of neuroinflammation of a novel 18F-labelled PET tracer targeting P2X7
Source: EJNMMI Res. 2017 Apr 4;7:31. doi: 10.1186/s13550-017-0275-2 (PMC5378566; doi:10.1186/s13550-017-0275-2)
Supplement: Supplementary file 1 — Supplementary materials. (DOCX 284 kb) [file 13550_2017_275_MOESM1_ESM.docx]

# Supplementary materials

## Molecular modelling

The homology model of the hP2X7 receptor was built using the X-ray structure of the close state zP2X4 receptor as a template (PDB code [4DW0](http://www.rcsb.org/pdb/explore.do?structureId=4DW0), 2.9 Å resolution). First, alignment of the zP2X4 and hP2X7 receptor primary sequences was performed within MOE. The primary sequences of the other human P2X subtypes were included in this step to improve the performance of the alignment analysis. The original zP2X4 receptor boundaries were applied for the corresponding hP2X7 sequence. The missing domains were built by the loop search method implemented in MOE. Once the heavy atoms were modelled, all hydrogen atoms were added and the protein coordinates were then minimized with MOE using the AMBER99 force field (47) until the root mean square (RMS) gradient of the potential energy was less than 0.05 kJ mol^−1^ Å^−1^. Reliability and quality of the model were checked using the Protein Geometry Monitor application within MOE.

The ligand structures were docked individually into the binding site of the P2X7 receptor using the AutoDock tool (PyrX interface) by the Scripps Institute (16, 17). Lamarckian genetic algorithm was employed for this analysis with the following settings: 50 runs for each ligand; 2,500,000 as maximum number of energy evaluations; 27,000 as maximum number of generations; 0.02 as rate of gene mutation and 0.8 as rate of crossover. The grid box was set with 50, 50, and 50 points in the x, y, and z directions, respectively, with the default grid spacing of 0.375 Å. The Binding_Energy parameter was used as an estimate of the variation in free energy (ΔG) arising from the docking. The docking conformations were then imported into MOE and the partial charges were assigned by semi-empirical RHF/AM1 calculations using the MOPAC package (18) implemented in MOE. All the generated ligand-receptor complexes were subjected to AMBER99 force field energy minimization in MOE until the RMS gradient of the potential energy was less than 0.05 kJ mol^−1^ Å^−1^. Receptor residues within 6 Å distance from the ligand were allowed to move, while the remaining receptor coordinates were kept fixed. AMBER99 receptor partial charges and MOPAC output ligand partial charges were utilized. Upon completion of the compound-binding site energy minimization, receptor coordinates were fixed and a second energy minimization with MMFF94 force field (48, 49) was performed allowing only the ligand atoms to move. For each ligand, the minimized docking poses were then rescored using the dock-pK_i_ predictor. This tool estimates the pK_i_ for each ligand using the empirical scoring function “scoring.svl” script retrievable at the SVL exchange service (Chemical Computing Group, Inc. SVL exchange: <http://svl.chemcomp.com>). The obtained pK_i_ values must be considered as docking scores and not as prediction of binding affinity. For each compound, the top-score docking poses according to at least two out of three scoring functions were selected for final ligand-target interaction analysis. The use of two different scoring parameters was deliberate and aimed at increasing the degrees of confidence in the output values.

## Chemistry and radiochemistry materials

Commercial grade reagents and solvents were purchased from Sigma Aldrich, Alfa Aesar, VWR International, Fisher Scientific Ltd., SLS and Santa Cruz Biotechnology and used without further purification, unless otherwise stated. All reactions, unless otherwise stated, were carried out at room temperature, with magnetic stirring and in a fume hood. Microwave heating was performed with a CEM Discover Analyser. Organic extracts were dried with anhydrous magnesium sulphate and evaporated using an ice cooled rotary evaporator. All synthetic products were dried under vacuum in a desiccator with activated anhydrous calcium carbonate, sealed and stored at 4°C under an inert atmosphere. All radioactive reactions were performed in 3 mL or 5 mL Wheaton V-vials, except for the reactions >110°C where 16 x 125 mm borosilicate tubes fitted with PTFE/silicone-lined screw caps (Sigma Aldrich).

### Spectroscopy

Infra-red spectra were recorded from solid substances on a Perkin-Elmer Spectrum 100 FT-IR spectrometer. Only selected absorbances (νmax) are reported. ^1^H NMR and ^13^C proton decoupled NMR spectra were recorded at 400 MHz in 5 mm tubes on Bruker AV-400 spectrometers. Spectra were analysed with ACD/NMR processor (academic edition), V12.01. Chemical shifts (δ_H_ and δ_C_, respectively) are quoted in parts per million (ppm) and referenced to the appropriate residual solvent peak.(50) Coupling constants (J) are reported to the nearest 0.1 Hz.

### Chromatography

Flash column chromatography (FCC) was performed on silica gel (Merck Kieselgel 60 F_254_ 230-400 mesh). Thin Layer Chromatography (TLC) was performed on aluminium-backed plates pre-coated with silica (0.2 mm, 60 F254) which were developed using UV fluorescence, vanillin, potassium permanganate, iodine or ninhydrin staining. Chemical and radiochemical analysis and purification was performed manually on an Agilent 1200 series quaternary pump HPLC, coupled to a UV detector (254 nm) in series with a FC-3200 NaI/photomultiplier tube (PMT) LabLogic radiodetector. Radioactive TLCs were developed with the methods specified in the text and analysed on a Bioscan Flow-Count TLC scanner equipped with a FC-3600 Plastic Scintillator/PMT LabLogic detection probe. HPLC mobile phase gradients are specified in the text. Spectra were recorded and analysed with Laura 4 (LabLogic) or GINA Star 5.8 (Raytest). Chemical mass analysis was performed on an Agilent 1200 HPLC with an Agilent 6520 Accurate Mass QTOF LC/MS with ESI. The instrument was equipped with an autosampler, UV detection (254 nm) and samples were injected directly or through a Phenomenex Kinetex XB-C18 4.6 mm x 150 mm 5 μm column. Spectra were recorded and analysed with MassHunter Workstation 2009 B.02.01 and Analysis 2009 B.03.01, respectively. HPLC column specifications: Kinetex Phenomenex XB-C18 with guard, 4.6 x 150 mm, 5 μm, 100 Å; Agilent Eclipse XDB C18, 9.4 x 250 mm, 5 μm, 100 Å. Solid phase extraction (SPE) cartridge specifications: Sep-Pak light lC18 Short SPE, 130 mg, 55-105 µm (cat. no. WAT023501, Waters), Sep-Pak tC18 Plus Long SPE Cartridge, 900 mg, 37-55 µm (cat. no. WAT036800, Waters), Sep-Pak Accell Plus QMA SPE Cartridge, 130 mg, 37-55 μm (cat. no. WAT023525, Waters), Strata-X-CW, 200 mg/3 mL, 33 μm (cat. no. 8B-S035-FBJ, Phenomenex).

### Stability

| Time = 0 min | Time = 120 min |
| --- | --- |

Supp. Fig. 1 Stability of **[^18^F]EFB** in a 0.05% DMSO solution at 37.5°C, visualised by HPLC method B elution at 254 nm. UV_A: UV chromatogram; ChA: Channel A (Radiochromatogram); Cps: counts per second.

## Chemical synthesis methods

### Compound **4** – 4-Cyano-N,N,N-trimethylanilinium tri-fluoromethanesulfonate

Similarly to Haka *et al.* (51), 4-(dimethylamino)-benzonitrile (2.43g, 16.6 mmol) and methyl trifluoromethanesulfonate (2.1 mL, 18.3 mmol) were dissolved in DCM (30 mL) under an inert atmosphere. Upon overnight stirring at room temperature under argon, the solution turned cloudy. The precipitate was collected by vacuum filtration, washed with ethyl acetate and recrystallized in MeOH/EtOAc to give the product as pale yellow crystals in 10% yield (0.562 g). ^1^H NMR (400 MHz, D_2_O): 8.01 (s, 4H, H_1_-H_2_), 3.65 (s, 9H, H_3_). ^13^C NMR (100 MHz, D_2_O): 134.67, 121.17, 56.84. IR (ν, cm^-1^): 2242 (C≡N), 1500 (^+^N-R_4_), 1260, 1161 (SO_2_-O), 1025 (C-F).

The synthesis of compounds **2, 6** and **EFB** was carried out on the lines of previously published methods by Donnelly-Roberts *et al.* (8) and Carroll *et al.* (11) with the following modifications.

### Compound **2** – 5-isothiocyanatoquinoline

**1**

A solution of 5-aminoquinoline **1** (0.72 g, 5.0 mmol) in dichloromethane (15 mL) and saturated aqueous sodium bicarbonate (1.47 g in 15 mL, 17.25 mmol) at 0°C was added dropwise to a solution of thiophosgene (0.38 mL, 5.0 mmol) in dichloromethane (3 mL) and stirred for 2 h. The biphasic mixture was separated with more saturated sodium bicarbonate (10 mL) and dichloromethane (3 x 10 mL). Then the organic layer was washed with brine (2 x 15 mL), dried with MgSO_4_ and concentrated under reduced pressure. Purification was carried out with flash column chromatography (SiO_2_, 25%-50% EtOAc in hexane). The pale yellow powder isolated in 62% yield, 0.58g (3.1 mmol) was dried and characterised as 5-isothiocyanato-quinoline (compound **2**). ^1^H NMR (400 MHz, CDCl_3_) δ (ppm): 9.00 (dd, 1H, H_1_, J = 4.3 Hz, 1.6 Hz), 8.47 (dd, 1H, H_3_, J = 8.5 Hz, 1.6 Hz), 8.07 (dd, 1H, H_4,_ J = 8.6 Hz, 1.0 Hz), 7.68 (dd, 1H, H_5,_ J = 8.6 Hz, 7.5 Hz), 7.55 (dd, 1H, H_2,_ J = 8.5 Hz, 4.3 Hz), 7.51 (dd, 1H, H_6_, J = 7.5 Hz, 1.0 Hz). ^13^C NMR (100 MHz, CDCl_3_) δ: 151.45, 131.24, 129.23, 129.00, 123.93, 122.08. HRMS (ESI^+^) m/z 187.0492 (M+H)^+^. IR (ν, cm^-1^) 2129 (N=C=S).

### Compound **6** – Methyl N'-cyano-N-5-quinolinylcarbamimidothioate

^1^H NMR (400 MHz, CDCl_3_) δ (ppm): 9.02 (dd, 1H, H_1_, J = 4.2 Hz, 1.6 Hz), 8.74 (br s, H_7_), 8.32 (dd, 1H, H_3_, J = 8.5 Hz, 1.6 Hz), 8.22 (dd, 1H, H_6_, J = 8.5 Hz, 1.1 Hz), 7.77 (dd, 1H, H_5_, J = 8.5 Hz, 7.4 Hz), 7.59 (dd, H_4_, J = 7.4 Hz, 1.1 Hz), 7.55 (dd, H_2_, J = 8.5 Hz, 4.2 Hz), 2.38 (s, 3H, H_8_); ^13^C NMR (100 MHz, CDCl_3_) δ (ppm): 151.36, 131.52, 130.71, 128.80, 127.19, 122.30, 14.59. IR (ν, cm^-1^) 3195 (N-H), 2161 (C≡N), 1509 (N-C=S), 1490 (N-C-N). MS (ESI^+^) m/z 243.0695 (M+H)^+^. Elemental Analysis: Calc. For C_12_H_10_N_4_S C, 59.48; H, 4.16; N, 23.12; S, 13.23; found: C, 59.37, 59.42; H, 4.14 4.22; N, 22.96 23.03.

### **EFB** – 2-Cyano-1-(4-fluorobenzyl)-3-(quinolin-5-yl)guanidine

^1^H NMR (400 MHz, MeOD) δ (ppm): 8.94 (dd, 1H, H_1_, J = 4 Hz, 1.6 Hz), 8.29 (dd, 1H, H_3_, J = 8 Hz, 1.6 Hz), 8.17 (d, 1H, H_4_, J = 8.6 Hz), 7.88 (dd, 1H, H_5_, J = 8 Hz, 8.6 Hz), 7.67 (d, 1H, H_6_, J = 8 Hz), 7.64 (dd, 1H, H_2_, J = 8 Hz, 4 Hz), 7.37 (dd, 2H, H_11_, J = 5.4 Hz, 7.7 Hz), 7.07 (dd, 2H, H_10_, J = 5.4 Hz, 7.7 Hz), 6.96 (s, 1H, H_8_), 4.47 (s, 2H, H_9_). HRMS (ESI^+^): calcd. for C_16_H_15_FN_8_ (M+H) 339.1477, found 339.1501; calcd. for (M+Na) 320.1306, found 320.1307. IR (ν, cm^-1^): 2181 (C≡N), 1706 (guanidine C=N), 1509 (N-CN), 1025 (C-F).


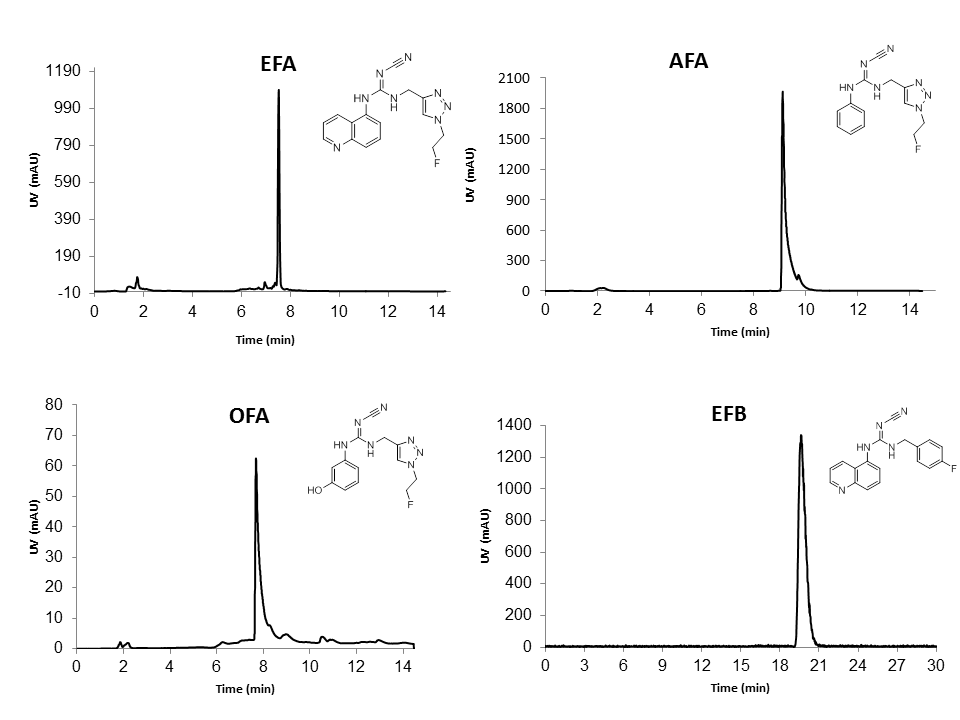


Supp. Fig. 2 Purity of >99% was confirmed by UV-HPLC using method B at 300 nm (Table 1 in main text).

## *In vitro* assays

### Materials

Buffers: heat inactivated foetal bovine serum (FBS, Sigma), DMEM-F12 (Sigma-Aldrich). Antibiotics: Penicillin (Invitrogen, Italy), Streptomicin (Invitrogen, Italy). Cell harvesting: Trypsin-EDTA solution (Sigma). Transfection reagents: pcDNA3 plasmid (Invitrogen), G418 sulfate (Geneticin). Assays: Fura-2/AM (Molecular Probes, Inc., Eugene, OR, USA), 2′(3′)-O-(4-Benzoylbenzoyl)adenosine 5′-triphosphate triethylammonium salt (BzATP, Sigma).

### Cell cultures and transfections

HEK293 and B16 cells were cultured in DMEM-F12 complemented with 10% heat-inactivated foetal bovine serum (FBS), 100 U/ml penicillin, and 100 mg/ml streptomycin. Cells were transfected with the calcium phosphate method as described in Rizzuto *et al.* (21) and Morelli *et al.* (22) All cDNAs were in pcDNA3 plasmid. Stably P2X7 transfected, single cell-derived clones were obtained by limiting dilutions. Transfected cells were then kept under selection in the presence of 0.2mg/ml G418 sulfate. Surface expression of P2X7 was performed by indirect immunofluorescence and flow cytometry with a P2X7 mouse monoclonal antibody directed against the extracellular domain of the human P2X7 receptor.

## Preliminary *in vivo* evaluation

Suppl. Fig. 3 Percent weight variation 24 hours post-treatment in 0.32±0.05 mL, 0.5 mg/kg LPS treated versus 0.32±0.05 mL saline-injected control rats. Veh: vehicle (saline) injection.

## PET/CT additional methods

### Anaesthesia

Dynamic PET imaging of all rats was performed under isoflurane anaesthesia. The oxygen was kept at 1 L/min flow rate with isoflurane levels of 5% for induction and termination, while for maintenance the isoflurane rate was set to 1.5-3% with constant breathing and temperature monitoring.

### PET/CT acquisition and quantitative image analysis

PET/CT scans were acquired with Nuclide^TM^ within the energy window of 400-600 keV, coincidence detection ratio 1-5 and voxel resolution of 0.30 x 0.30 x 0.30 mm^3^. CT scanning was performed immediately before or after the PET scans with X-ray energy of 45 KVp, 360 projection and 0.25 x 0.25 x 0.21 mm^3^ voxel size. The datasets were resolved by dynamic fine reconstructions with coincidence detection ratio 1-5 and decay correction to the time of injection. Binning was performed in incremental time units of 5 to 30 min size. All reconstructed datasets were analysed with VivoQuant 1.21 (InviCRO, LLC, Boston, USA). ROI activity quantification was corrected for background noise. Uptake was expressed as percent injected dose per gram of tissue (%ID/g), as calculated with reference to the injected activity dose at the time of injection and the ROI size in mm^3^. The blood ROI was sampled from the coronary artery. All data are presented as mean ± SEM unless otherwise stated.

### Biodistribution

Immediately after imaging, the animals were culled by terminal anaesthesia and cervical dislocation. The animals were dissected and organs and body fluids were collected for gamma counting (LKB Wallac 1282) and weighed. An ^18^F counts-to-becquerel standard curve was made so as to enable conversion between the two units. The radiotracer uptake in each organ was expressed as %ID/g, where %ID was the percent ratio of the measured organ radioactivity to the total administered dose. The latter was defined as the MBq injected to the animals, decay corrected to the start of counting.
